# Scared to Trust? – Predicting Trust in Highly Automated Driving by Depressiveness, Negative Self-Evaluations and State Anxiety

**DOI:** 10.3389/fpsyg.2019.02917

**Published:** 2020-01-23

**Authors:** Johannes Kraus, David Scholz, Eva-Maria Messner, Matthias Messner, Martin Baumann

**Affiliations:** ^1^Department of Human Factors, Institute of Psychology and Education, Ulm University, Ulm, Germany; ^2^Department of Clinical Psychology and Psychotherapy, Institute of Psychology and Education, Ulm University, Ulm, Germany; ^3^Department of Clinical and Health Psychology, Institute of Psychology and Education, Ulm University, Ulm, Germany

**Keywords:** trust in automation, anxiety, depressiveness, personality, self-evaluation, emotional states, trust formation, automated driving

## Abstract

The advantages of automated driving can only come fully into play if these systems are used in an appropriate way, which means that they are neither used in situations they are not designed for (misuse) nor used in a too restricted manner (disuse). Trust in automation has been found to be an essential psychological basis for appropriate interaction with automated systems. Well-balanced system use requires a calibrated level of trust in correspondence with the actual ability of an automated system. As for these far-reaching implications of trust for safe and efficient system use, the psychological processes, in which trust is dynamically calibrated prior and during the use of automated technology, need to be understood. At this point, only a restricted body of research investigated the role of personality and emotional states for the formation of trust in automated systems. In this research, the role of the personality variables depressiveness, self-efficacy, self-esteem, and locus of control for the experience of anxiety before the first experience with a highly automated driving system were investigated. Additionally, the relationship of the investigated personality variables and anxiety to subsequent formation of trust in automation was investigated. In a driving simulator study, personality variables and anxiety were measured before the interaction with an automated system. Trust in the system was measured after participants drove with the system for a while. Trust in the system was significantly predicted by state anxiety and the personality characteristics self-esteem and self-efficacy. The relationships of self-esteem and self-efficacy were mediated by state anxiety as supported by significant specific indirect effects. While for depression the direct relationship with trust in automation was not found to be significant, an indirect effect through the experience of anxiety was supported. Locus of control did not show a significant association to trust in automation. The reported findings support the importance of considering individual differences in negative self-evaluations and anxiety when being introduced to a new automated system for individual differences in trust in automation. Implications for future research as well as implications for the design of automated technology in general and automated driving systems are discussed.

## Introduction

It is announced that in the next years, highly automated vehicles will become an affordable, everyday technology with a broad user group (Hörl et al., 2016). Commonly, the advent of automated vehicles is linked to positive outcomes like more efficient, safe, and comfortable driving (Kühn and Hannawald, 2015; Winkle, 2015). As the decision to use or not use driving automation will be made by human drivers in the next years, the associated advantages only come fully into play, if users decide to use this technology – and if so in an appropriate manner.

Trust in automation has been found to be a major subjective prerequisite for behavioral decisions in the interaction with various automated technological systems (e.g., Lee and Moray, 1994; Muir and Moray, 1996; Lewandowsky et al., 2000; Biros et al., 2004; Sanchez et al., 2004) and automated driving systems in specific (e.g., Dzindolet et al., 2003; Rajaonah et al., 2006; Hergeth et al., 2016; Payre et al., 2016). It is essential for safe and efficient use of automated driving that a calibrated trust level in accordance with a system’s capabilities and performance (e.g., Muir, 1987) is facilitated as it diminishes inappropriate system use. Conversely, if users’ trust is not calibrated, the dangers of disuse (underachieving the full potential of a system) and misuse (overstretching the capabilities of a system) of the system are increased (Parasuraman and Riley, 1997). To prevent these negative outcomes and in order to facilitate trust calibration, a thorough understanding of the psychological processes associated with trust formation and calibration is essential (e.g., Lee and See, 2004; Hoff and Bashir, 2015; Kraus et al., 2019b). Especially the relation of personality traits and emotional states to individual differences in trust in automation are a promising direction to gain an understanding for the underlying psychological mechanisms of trust formation and calibration (e.g., Hoff and Bashir, 2015). These processes could be addressed to personalize the design of automated technology in order to enhance trust calibration (e.g., prior information about the system, training, system functionality, and user interfaces).

Personality as a collection of “mechanisms within the individual that are organized and relatively enduring and that influence his or her interactions with […] environments” (Larsen and Buss, 2005, p. 4), might shape how different persons perceive and experience a technological system. For example, on a theoretical level, personality traits like risk aversion (Glöckner and Hilbig, 2012) should influence how potential costs and benefits of automated technology are weighted and interpreted. In a similar manner, personality traits in regard to the self-assessment of one’s abilities (e.g., self-efficacy; Bandura, 1991) might influence as how capable people see themselves to use new technology in a functional manner. In line with this general idea of a role of personality for trust formation, personality traits like neuroticism (Szalma and Taylor, 2011) and need for cognition (Kraus et al., 2019a) have been found to be associated with differences in trust in automation.

Furthermore, personality traits play an important role in the individual tendency to experience emotional states (e.g., Larsen and Ketelaar, 1991; Letzring and Adamcik, 2015). For example, the link between neuroticism and the tendency to experience negative affect is well supported (e.g., Larsen and Ketelaar, 1991). Emotional states have also been found to predict differences in trust in automation. For example, Stokes et al. (2010) found higher trust levels in persons experiencing positive affect than those experiencing negative affect.

Another theoretically meaningful emotional state for the explanation of individual differences in trust in automation is the experience of anxiety in the face of new technology. Anxiety might prevent rational choice in interactions with new technology and result in inefficient or even dangerous decisions (Peters et al., 2006). Such irrational decision-making would result in a reduction of an overall positive effect of the advantages of automated driving (e.g., increased road safety and more efficient and economic traffic system; see for example Kühn and Hannawald, 2015; Winkle, 2015). In line with this, in their review on interpersonal trust, Thielmann and Hilbig (2015) theoretically link the individual experience of anxiety (trait anxiety) to trust behavior. At this point, the relation between state anxiety and trust in automation has not yet been investigated.

**FIGURE 1:**
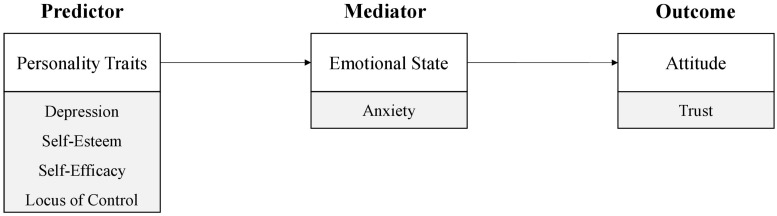
Investigated relationship of personality, state anxiety and trust in automation. It is hypothesized that the relationship of depression, self-esteem, self-efficacy, and locus of control to initial learned trust in automation is mediated by the extent of an individual experience of anxiety at the time when a new and unfamiliar automated driving system is introduced.

In this research, the relationship between anxiety, when being introduced to a new automated driving system, and the initial level of learned trust after a first interaction with the system is investigated in a driving simulator study. Furthermore, the role of the personality variables depressiveness (Beck et al., 1961), self-esteem, self-efficacy and locus of control (e.g., Chamorro-Premuzic et al., 2008; Xiao et al., 2014), for the experience of anxiety and individual differences in trust in automation is investigated. The underlying idea of this research is that depressiveness and negative self-evaluations lead to a more anxious perception of and approach to an unfamiliar technological system. The resulting increased level of anxiety leads to decreased trust levels in the system. More specifically, the underlying research hypothesis is that the effect of depressiveness, self-esteem, self-efficacy, and locus of control (LOC) on trust in automation is mediated by an experience of anxiety in the face of unfamiliar automated technology (see Figure 1). In the following, the theoretical background of the study is provided along with an integrated derivation of study hypotheses.

## Theoretical Background

### Factors Influencing the Calibration of Trust in Automation

Lee and See (2004) define trust in automation as “the attitude that an agent will help to achieve an individual’s goal in a situation characterized by uncertainty and vulnerability” (p. 51). The psychological process, in which trust in automation is built up prior and during the interaction with an automated system is subject to several theoretical reviews and frameworks (e.g., Lee and See, 2004; Schaefer et al., 2014; Hoff and Bashir, 2015) as well as empirical research (e.g., Beggiato et al., 2015; Hergeth et al., 2016; Kraus et al., 2019a, b). A multitude of factors within the system, the person and the situation have been linked to trust calibration processes (see Hancock et al., 2011 for an overview). On the side of the user, personality (e.g., Merritt and Ilgen, 2008; Merritt et al., 2015; Kraus et al., 2019a) as well as emotional states (e.g., Stokes et al., 2010; Merritt, 2011) have been found to influence trust formation and explain inter-individual differences in trust toward specific automated systems. In support of this notion, reviews and meta-analyses in human-machine interaction propose personality to be an important predictor for inter-individual differences in trust in automation (e.g., Lee and See, 2004; Hancock et al., 2011; Hoff and Bashir, 2015; Schaefer et al., 2016; Hock et al., 2018). To extend the understanding of the role of emotional states and personality for the dynamic formation of trust in automation, this research investigates the relationships of depressiveness, negative self-evaluations, and anxiety to learned trust in an automated driving system. The hypothesized mediation process of this research (Personality traits → emotional state → trust in automation) gained support in previous studies in the domain of interaction with technology (e.g., Jacques et al., 2009).

### Anxiety and Trust in Automation

Emotional states were defined as “a state of physiological arousal and of a cognition appropriate to this state of arousal” (Schachter and Singer, 1962, p. 380). The impact of emotional states on judgments and behavior has been subject to a great deal of research (e.g., Forgas and George, 2001; Dunn and Schweitzer, 2005; Forgas and East, 2008). Prominently, the affect-as-information model (Schwarz and Clore, 1988) proposes that people tend to misattribute a momentary experienced mood to their judgments by asking themselves how they feel about an object of evaluation. A second model proposing effects of emotional states to judgments is the affect infusion model (AIM; Forgas, 1995). Based on these theories, Dunn and Schweitzer (2005) propose that emotional states might be used as an important source of information for building up interpersonal trust. In line with this reasoning, emotional states were empirically found to be related to trust in automated systems (e.g., Merritt, 2011). Stokes et al. (2010) showed that the impact of emotional states on trust in an automated system was strongest at the beginning of the interaction when the system is unknown and diminished with repetitive system use.

While prior research mostly focused on positive and negative affect (Stokes et al., 2010), this research investigates the relation of state anxiety to trust in automation. State anxiety is conceptualized as a momentary emotional state, which is accompanied by a consciousness feeling of tension and uneasiness and a higher activity of the autonomic nervous system (Spielberger, 1966; Horikawa and Yagi, 2012). Anxiety has been found to affect information processing of ambigiuous (e.g., Mathews and Mackintosh, 1998) and threatening stimuli (Mathews, 1990). Similarly, trusting behavior refers to a risky choice of depending on another party (e.g., Thielmann and Hilbig, 2015) in a situation characterized by uncertainty, vulnerability and the inability to control the actions of another party (e.g., Mayer et al., 1995; Lee and See, 2004). Therefore, the individual level of trust in automation should essentially be influenced by perceived risk and the associated experience of anxiety. In line with this, a theoretical link between the individual disposition to experience anxiety, risk/loss aversion and trust has been proposed for interpersonal contexts (e.g., Thielmann and Hilbig, 2015). Also, in the context of automated driving, anxiety has been investigated as a dependent variable additional to trust in automation. Du et al. (2019) report a significant negative relationship between trust and anxiety although this relationship was not in the focus of their research and anxiety was measured as an attitude instead of an emotional state.

In this research, state anxiety is hypothesized to be associated with decreased initial trust in an unfamiliar, newly introduced automated driving system. Anxiety might lead to an overrepresentation of potential risks in the assessment of automated driving systems and thereby reducing trust in the systems. Additionally, in line with the affect-as-information model and the AIM the experience of anxiety in the face of a new system might be misattributed to the judgment of restricted trustworthiness of the system and therefore lead to lower trust. In the interaction with new (and potentially risky) technology, the experience of increasing levels of anxiety might progressively prevent rational choice and result in inefficient or even dangerous decisions (Peters et al., 2006). The theoretical importance of anxiety for an understanding of the psychological mechanisms in the formation and calibration of trust in automation is further supported by its incorporation in theoretical models of technology use (e.g., Osswald et al., 2012) and empirical support for its role in the interaction with automated systems (e.g., Nomura et al., 2008). Based on this theoretical association between the experience of anxiety and trust formation and in line with predictions of the affect-as-information model and the AIM, it is hypothesized:

Hypothesis 1 (H1): Higher state anxiety at the time when an unfamiliar automated driving system is introduced is associated with lower trust in the system.

Personality traits have been linked to the experience of emotional states in a long tradition of psychological research (e.g., Larsen and Ketelaar, 1991; Letzring and Adamcik, 2015). In line with this, anxiety has been found to be predicted by inter-individual differences in personality variables – besides others by depression (Nutt, 1999) and self-evaluations (e.g., Chamorro-Premuzic et al., 2008; Xiao et al., 2014). To gain a better understanding of the emergence of anxiety differences when being introduced to or initially interacting with unfamiliar automated technology, we investigated the role of these personality variables for individual differences in the extent of anxiety when being introduced to a new automated system.

### Depression and Trust in Automation

Depression is one of the most widespread psychological disorders in many societies. For example, in the United States, Major Depressive Disorder (MDD) is with a life-time prevalence of about 17% and a 12-months-prevalence of 7% (Kessler et al., 2005a) one of the most prevalent mental disorders (Kessler et al., 2005b). Not only clinical depression is affecting individuals, but subclinical depression is also widely common (Horwath et al., 1992; Cuijpers et al., 2004). An individual is referred to as subclinical depressed as he or she experiences clinically relevant depressive symptoms but does not meet the diagnostic criteria (e.g., Cuijpers et al., 2014).

As depressed individuals were shown to perceive and accept the use of technology differently from other user groups (e.g., Baumeister et al., 2014; Ebert et al., 2015), it is likely that also (subclinical) depressed persons show different attitudes toward the use of technology as compared to non-depressed individuals. Depression is characterized by a lack of interpersonal trust (Lester and Gatto, 1990; Kim et al., 2012) and this inability to build trust to other people might impair the ability to build trust into an automated driving system as well. In Hoff and Bashir’s model of trust in automation (2015) it is stated that the internal variability in trust is rooted in (1) self-confidence, (2) subject-matter expertise, (3) mood, and (4) attention capacity. In (subclinical) depression, besides others self-confidence, mood, psychomotoric abilities, decision-making and concentration are distorted (e.g., Fried et al., 2016; Fried, 2017) and therefore, the ability to build trust in automated systems might be impaired. We, therefore, hypothesize that depressiveness (the amount of experiencing depression symptoms) is associated with lower trust in an automated driving system:

Hypothesis 2 (H2): Depressiveness is negatively related to trust in an automated driving system.

### Mediation of the Relation Between Depressiveness and Trust in Automation by Anxiety

The experience of depressive symptoms is commonly associated with increased anxiety (e.g., Murphy et al., 2004; Cummings et al., 2014). The same holds true for subclinical depression (e.g., Lovibond and Lovibond, 1995). In fact, self-report questionnaires for depression and anxiety show strong correlations in both clinical and sub-clinical samples (Sowislo and Orth, 2013). Following from this association, the more depressive symptoms someone experiences the more anxious he or she should be when being introduced to a new technological system and accordingly trust an unfamiliar automated system less. The relation of depression and anxiety to dysfunctional interaction with technology has been already investigated in regard to smartphone addiction (Elhai et al., 2016). In accordance, it is hypothesized:

Hypothesis 3 (H3): The effect of depressiveness on trust in an automated driving system is mediated by the experience of anxiety at the time of introduction of the system.

### Traits Related to Negative Self-Evaluation and Trust in Automation

Besides depressiveness, personality traits related to negative self-evaluations were found to influence trust in other persons (e.g., Chamorro-Premuzic et al., 2008; Xiao et al., 2014). Also, for trust in automation, a relevance of negative self-evaluations is supported by the strong empirical evidence for the effect of situational self-confidence on differences in trust in automation (e.g., Lee and Moray, 1992, 1994; Moray and Inagaki, 2000). Self-confidence was conceptualized as a self-assessment of the capability to sucessfully perform a task with a technical system (e.g., Briggs et al., 1998). In this research, the role of self-esteem, self-efficacy, and LOC, which play an essential role in the emergence of self-confidence judgments, for individual differences in initial trust in an automated driving system is investigated.

Harter (1993) defined self-esteem as “the level of global regard one has for the self as a person” (p. 88) affecting how worthy, capable and successful people perceive themselves to be (Coopersmith, 1981, cited in Herz and Gullone, 1999). Self-esteem has been shown to be related to self-confidence (e.g., Owens, 1993).

Similarly, self-efficacy builds a personality basis for situational self-confidence and can be understood as a trait describing a broad, trans-situational form of self-confidence in one’s ability to perform well in various tasks (e.g., Locke et al., 1984; Schunk, 1991; Locke and Latham, 1994). General self-efficacy was defined by Bandura (1991) as “people’s beliefs about their capabilities to exercise control over their own level of functioning and over events that affect their lives” (p. 257).

The mentioned evidence for the relationship of trust in automation and situational self-confidence points into the direction that both self-esteem and self-efficacy (as personality facets affecting self-confidence) influence the self-evaluation of one’s own ability to successfully interact with automated systems. Therefore, people with comparably high levels of self-esteem and self-efficacy should perceive themselves as capable of using this technology safely and in line with their needs and goals and thus trust it more.

Hypothesis 4 (H4): Self-esteem is positively related to trust in an automated driving system.

*Hypothesis 5 (H5): Self-efficacy is positively related to trust in an automated driving system*.

Locus of control describes the disposition to perceive oneself as capable of controlling the outcome of an event or action (e.g., Rotter, 1966, 1990; Ajzen, 2002). The trait is formed by two antagonistic factors – internal (perception of an own ability to control events) and external LOC (outcomes are beyond own control). In line with Phares (1976), internals show more effort to control their fate, seek more information and use it better. In turn, several studies showed that internals are more efficient problem solvers (e.g., DuCette and Wolk, 1973). As a consequence, internals, through their decision-making, increase the likelihood that their experience is in line with their internal view of things (Spector, 1982). In contrast, an external locus of control has been related to learned helplessness (Seligman, 1972; Hiroto, 1974; Cohen et al., 1976). In line with these findings, it can be concluded that people with an internal LOC should be more willing to trust automation as they perceive themselves to be more in control of making functional decisions leading to positive results as compared to those with an external locus of control who do not perceive that they can control their fate. The role of LOC in the domain of driving has been investigated before with conflicting results (e.g., Özkan et al., 2005). Based on our theoretical reasoning, the following hypothesis is formulated:

Hypothesis 6 (H6): An internal locus of control (H4.3) is positively related to trust in an automated driving system.

### Mediation of the Relation Between Negative Self-Evaluations and Trust in Automation by Anxiety

Self-esteem is associated with an increased feeling of safety and security and serves an anxiety-buffering function (e.g., Greenberg et al., 1992), which is a central assumption of the Terror Management Theory (e.g., Greenberg et al., 1986). In a meta-analysis by Sowislo and Orth (2013), a negative correlation between self-esteem and anxiety was supported both for measuring the constructs simultaneously and in a longitudinal manner. Also, in regard to an unfamiliar automated system, people with a higher level of self-esteem should feel more secure about themselves and thus experience less anxiety of interacting with the system and trust it more in the subsequent interaction. Therefore, it is hypothesized that self-esteem is negatively related to an experience of anxiety when being introduced to a new automated system and that state anxiety mediates the positive relationship of self-esteem to learned trust in automation (H7.1).

For self-efficacy anxiety plays a central role (e.g., Bandura, 1988) as a threat is not merely determined by external dangers of a situation but rather a combination from the situation and both an assessment of dangers and perceived coping capability. Consequently, lower self-efficacy is hypothesized to be associated with a higher degree of anxiety in the face of a forthcoming interaction with an unfamiliar technological system, whereas anxiety mediates the positive effect of self-efficacy to trust in such a system (H7.2). In support of this hypothesis, bivariate correlations (with small to medium effect sizes) between computer self-efficacy, trust, and internet anxiety have been reported (Thatcher et al., 2007).

Third, LOC has consistently been found to be related to anxiety in the way that internals experience less anxiety than externals (e.g., Spector, 1982). It can be followed that a person with an internal LOC should experience less anxiety when being introduced to a new technology he or she did never interact with before as compared to a person with external LOC. The latter will tend to perceive this situation beyond his or her control and consequently be more anxious about its consequences. Accordingly, it is hypothesized that an internal LOC is associated with less and an external LOC with higher anxiety when being introduced to a new driving automation system. Subsequently, internals should trust such a system more than externals and this relationship is mediated by anxiety (H7.3).

Following from this, in this study it is hypothesized, that the relationships of the investigated personality variables to trust in automation are mediated by the experience of anxiety when the automated system is first introduced. Support for this mediation mechanism of the influence of traits through the experience of affective states has been found in other studies in the domain of technology interaction as well (e.g., Jacques et al., 2009).

Hypothesis 7 (H7): The effects of self-esteem (H7.1), self-efficacy (H7.2) and locus of control (H7.3) on trust in an automated driving system are mediated by the experience of anxiety at the time of introduction of the system.

The hypotheses were investigated in a driving simulator study with a correlative research design. Depression and personality traits were measured before an automated driving system was introduced. Also, state anxiety of participants was assessed, together with positive and negative affect as control variables to investigate the incremental effect of anxiety on trust in automation. Subsequently, participants were introduced to the system and drove with the automation in the driving simulator and evaluated their trust in the system afterwards.

## Materials and Methods

This study investigates the relationship of depression, self-esteem, self-efficacy, LOC, and the level of anxiety when being introduced to a new automated driving system to trust in the system in a correlative design. Participants answered questionnaires with the personality and state scales and were then introduced to an automated driving system. After a practice trial in manual mode to get used to the driving simulator, participants interacted with the automated driving system they had been introduced to earlier for about 5 min. Afterwards, participants rated their trust in the system.

### Sample

Participants were recruited at Ulm University with mailings and posters and were compensated with 10€ or study credit for their participation. After exclusion of two participants (automation error and non-compliance with study instructions), the final sample consisted of *N* = 47 participants, of which 27 were female (57%). Participants were on average *M* = 27.45 years old (*SD* = 9.75) and held their driving license for *M* = 9.38 years (*SD* = 9.16).

### Procedure

The study was conducted in the driving simulator lab at the Human Factors department at Ulm University. After being welcomed, a brief study introduction was given and informed consent was obtained from all participants. Participants received a series of personality questionnaires and questionnaires regarding their emotional states (see below). Afterward, they were introduced to the driving simulator, settings were adjusted, and participants were introduced to the automated driving system and the driving task. The system was introduced with a manual and an automated mode, while the latter enables automated driving on the motorway under certain conditions. In a practice trial participants could familiarize with the settings of the simulator and driving under these conditions in manual mode. After 4–5 min, they were asked by the investigator if they felt safe with driving in the simulator. If they indicated to not feel safe, they could continue the practice for a while, otherwise the study procedure continued. In the instruction text and in the practice trial, transparency of the automation was manipulated in two level (low vs. high transparency), which was not in the scope of this research (Kraus et al., 2019b). Afterwards, participants were instructed that they would now drive with the automated driving system; they had been introduced to earlier. After this instruction, the participants used the automated driving system for the first time in a test drive of about 5 min. Participants were instructed to adhere to traffic rules and not exceed a speed limit of 130 kilometers per hour. The test drive started at the entrance of a two-lane motorway and the vehicle was set in manual mode for about 1 min. Afterward, participants were instructed to activate the automated mode. In the course of the remaining time, participants went through several automated overtaking maneuvers. In between the system conducted a take-over request and asked the participant to drive manually again for a while due to road works. After the latter automated driving was indicated to be available again and participants were asked to reactivate automated driving. During the simulated drive, an attention assist was implemented in the simulated drive, which impelled participants with a repeated beep sound (every 60 s) to put the hands on the steering wheel and to direct their gaze on the road. Manual control could be taken over at any time. Half of the participants experienced a malfunction of the system during the test drive as part of a second manipulation also not in the scope of this research. Data analysis was conducted with the complete sample and the trust measurement after the test drive was used as the outcome variable (test of preconditions below).

The original study procedure continued afterward with a subsequent experimental drive interrupted by several questionnaires, which was part of another study reported elsewhere. After this drive, participants answered further questionnaires, amongst others on their demographics. Subsequently, participants were compensated for their participation, debriefed, and dismissed. In total, the study took 75 min. The reported research complied with the Declaration of Helsinki.

### Materials

#### Apparatus

**FIGURE 2:**
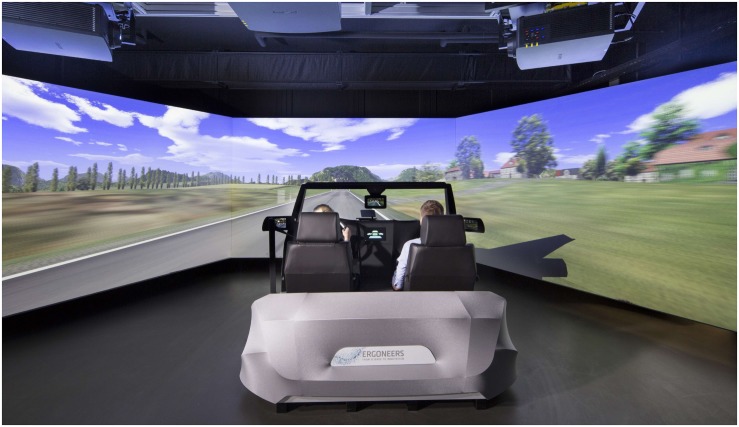
Driving simulator of the Human Factors Department at Ulm University. Photo by: H. Grandel/Ulm University.

The study was conducted in the driving simulator of the Human Factors department at Ulm University (see Figure 2). The simulator is equipped with a car interior mock-up with basic design elements and a windshield frame. For front and side view three 1920 × 1200px video projectors fill three screens of 3.3 × 2.1 m each and realize a 200° viewing angle. Rearview is implemented by two displays replacing the side mirrors and one display replacing the rearview mirror (each 7” with 800 × 400px; 16:9). For this study, an information display was located in the center console indicating the current state of the system and equipped with a touch interface to engage and disengage the automation.

#### Questionnaires

Depressiveness was assessed with the German questionnaire Becks Depressions Inventory II (BDI-II, Hautzinger et al., 2009), which covers thoughts, feelings, behaviors, and symptoms of a major depression with 21 items to be answered on a four-point Likert scale. LOC was investigated with the locus of control Scale (TOR-IE; Rotter, 1966) with 23 items reflecting both internal and external LOC. Higher values on this scale indicate a more external LOC. Self-esteem was assessed with the Rosenberg Self-Esteem Scale consisting of 10 items (Ferring and Filipp, 1996). Self-efficacy was measured with the German version of the General Self-Efficacy Scale (GSE, Jerusalem and Schwarzer, 1999). For the assessment of positive and negative affect, the Positive and Negative Affect Schedule (PANAS, Krohne et al., 1992) was used measuring the current affect with ratings on ten positive and ten negative adjectives with a five-point Likert scale. State anxiety was measured with a translated short version of the State-Trait Anxiety Inventory (STAI-6, Marteau and Bekker, 1992), in which participants had to assess their current feelings with six statements. Trust in automation was assessed with a German shortened version of the Automation Trust Scale (ATS; Jian et al., 2000) with seven items (see also Kraus et al., 2019b). The scale was designed to provide an economic measurement of trust in automation as a unidimensional construct. Scale means, standard deviations, Cronbach’s α along with the correlations of the scale values can be investigated in Table 1.

**TABLE 1:** Aggregated scale values (e.g., means or sum scores according to respective scale logic) standard deviation, internal consistencies (Cronbach’s α) and correlations of the investigated personality and state variables and trust in automation (correlations are based on scale means).

		*Score*	*SD*	α	1	2	3	4	5	6	7
1	BDI	4.98	5.92	0.881	–						
2	LOC	10.28	4.11	0.732	0.172	–					
3	Self-esteem	23.38	4.91	0.889	−0.622**	–0.179	–				
4	Self-efficacy	30.21	4.22	0.872	−0.481**	−0.446**	0.618**	–			
5	STAI	35.60	7.29	0.704	0.466**	0.315*	−0.606**	−0.489**	–		
6	PA	33.32	5.73	0.843	–0.055	–0.166	0.171	0.297*	–0.106	–	
7	NA	12.62	2.64	0.696	0.312*	0.212	−0.540**	−0.412**	0.400**	–0.135	–
8	Trust	5.26	0.99	0.880	–0.262	–0.218	0.370*	0.291*	−0.400**	0.229	–0.107

BDI = depressiveness; LOC = locus of control; STAI = state anxiety; PA = positive affect; NA = negative affect; Higher values in LOC indicate an external LOC; *p < 0.05; **p < 0.01.

### Statistical Procedure

Statistical analysis was conducted with R (version 3.4.3) and the R package lavaan (Rosseel, 2012; version 0.6–2). To test the hypothesized relationships, bivariate Pearson correlations were inspected (H1, H2, H4, H5, and H6) and path modeling was utilized (H3 and H7). For the path models, as recommended by Preacher and Hayes (2008), the residuals of constructs at mediating variable levels (states) were allowed to covary to avoid biased standard errors and model misspecification. For testing the study hypotheses, direct and specific indirect effects were investigated. Direct effects test the relationships between adjacent variables and indicate how well a variable directly predicts another variable (e.g., personality variable → emotional state). Indirect effects test the hypothesized mediations. The two types of effects were investigated with the percentile bootstrap procedure, as recommended by Hayes (2018), providing a 95% confidence interval (CI) for the estimations. The null hypothesis was rejected if zero was not included in the CI (Taylor et al., 2008; Hayes, 2009). We used 5000 bootstrap samples as recommended by Hayes (2009). In the following, standardized direct and indirect effects are reported.

Due to the mentioned manipulation of an independent variable (transparency; not in the scope of this study) in between the measurement of the personality and state variables and the measurement of trust in automation, to rule out any biases by group effects (interaction of the traits/states and the independent variable), a series of general linear models was conducted. As no such interaction was found to be significant, all reported statistics and indices can be viewed as unbiased by the experimental manipulations and can therefore be interpreted without problems.

Concerning the preconditions of the applied statistical procedures, one participant was identified as a univariate outlier for both depressiveness and self-esteem (|z| > 3.29) as well as a multivariate outlier as indicated by Mahalanobis distance. A closer inspection of the answering pattern showed a BDI score of 37 and a self-esteem score of seven for the individual, both values in a valid range indicating depression. As no further reason for exclusion could be identified and an exclusion of this participant would lead to an invalid restriction of the sample variance we did not exclude the participant. Furthermore, several of the investigated variables were not normally distributed as indicated by significant Shapiro–Wilk tests (depression, self-esteem, self-efficacy, negative affect and trust). As the Pearson correlation coefficient has been shown to be robust to violations of normality (e.g., Havlicek and Peterson, 1976; Bishara and Hittner, 2012) the reported correlations the reported correlations are valid to be interpreted. In the same way, the reported bootstrapped effects of the path models are not affected by distribution assumptions.

## Results

### Relationship Between Emotional States and Trust in Automation

**FIGURE 3:**
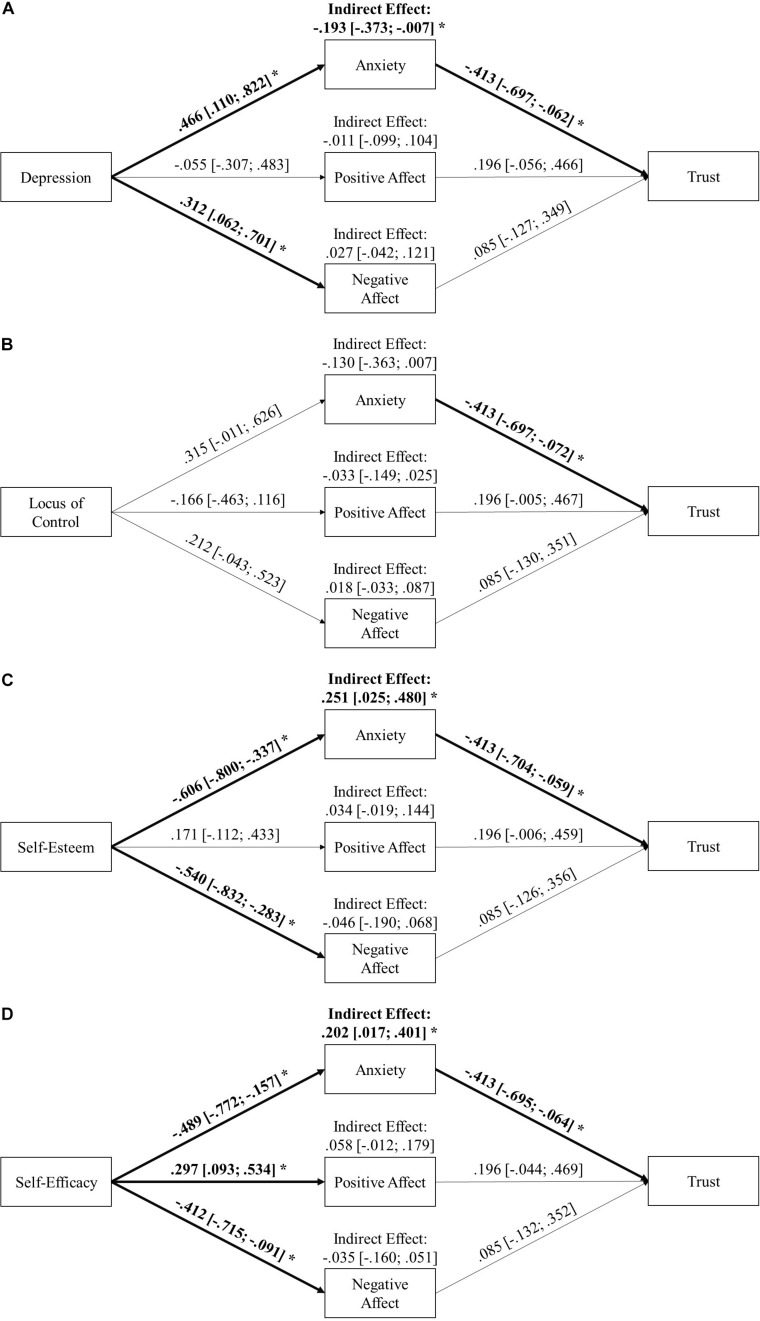
Standardized direct and indirect effects of the personality variables on trust in automation mediated by the three emotional states. Separate path models were calculated for each personality variable (**A**: Depression, **B**: Locus of Control, **C**: Self-Esteem, **D**: Self-Efficacy). Significant effects (α = 0.05) are indicated by an asterisk.

Hypothesis 1 stated that higher levels of anxiety are related to lower trust in an automated driving system. Inspection of the bivariate correlation of state anxiety and trust in automation revealed a strong negative correlation of *r* = −0.400 (*p* < 0.001) between state anxiety and trust in automation in support of H1 (see Table 1). Also, in terms of the investigated path models, anxiety showed a significant negative direct effect on trust in automation, β = −0.413 [−0.697; −0.062] (CIs from the model with Depression, Figure 3A), likewise supporting H1. To evaluate the extent of incremental variance of anxiety in the explanation of trust in automation above those emotional states already investigated (Stokes et al., 2010), correlations between trust in automation and positive and negative affect were inspected (see Table 1). Although in the same direction as in earlier research, both correlations were found to be non-significant in this study. Neither did both states mediate the effect of the investigated personality variables on trust in automation as indicated by non-significant indirect effects in all analyses (see Figures 3A–D). Taken together, in this study anxiety predicts trust in automation better than both positive and negative affect.

### Relationships Between Personality Variables and Trust in Automation

Also, hypotheses on the direct effects of the investigated personality variables on trust were tested with bivariate correlations (see Table 1). Regarding H2, the analysis revealed that depression was not directly related to trust (*r* = −0.262; *p* > 0.05). However, in the face of the comparably strong effect size and the significant indirect effect of depression on trust through anxiety (see below, Figure 3A), H2 gains some support in this study. Regarding the hypotheses on the relationships of traits reflecting negative self-evaluations and trust in automation, both self-esteem (H4; *r* = 0.370; *p* < 0.05) and self-efficacy (H5; *r* = 0.291; *p* < 0.05) showed the hypothesized significant positive correlation with trust (see Table 1). The correlation between LOC and trust, however, did not reach significance, thus contradicting H6 (*r* = −0.218; *p* > 0.05).

### Mediation of the Effects of Personality Variables on Trust in Automation by State Anxiety

To test the hypothesized mediations of the effects of the investigated personality variables on trust in automation by state anxiety (H3 and H7), parallel-mediation models were computed separately for each trait (see Figure 3). Separate models were calculated to prevent underestimation of the true relationships as a result of intercorrelations of the investigated traits (Hayes, 2018). Positive and negative affect were included on the state level as control variables to investigate if state anxiety provides variance explanation in the outcome trust in automation additional to these two emotional states.

As a statistical test for H3 and H7, indirect effects were investigated. As shown in Figure 3, in line with the hypotheses the indirect effects of depression (H3; β = −0.193 [−0.373; −0.007]; Figure 3A), self-esteem (H7.1; β = 0.251 [0.025; 0.480]; Figure 3C) and self-efficacy (H7.2; β = 0.202 [0.017; 0.401]; Figure 3D) on trust in automation via state anxiety were found to be significant. For self-esteem and self-efficacy (see Figures 3C,D), this effect can be interpreted as a mediation, as the correlation between self-esteem and trust is significant on their own (see Table 1). For depression, interestingly (see Figure 3A), this finding has to be interpreted as a mere indirect effect as the direct correlation between the trait and trust was not significant (see Table 1). In the same way as LOC did not show any effect on state anxiety in the first place, no significant indirect effect on trust in automation could be observed, which is in contradiction to H7.3 (β = −0.130 [−0.363; 0.007]; see Figure 3B).

### Relative Importance of the Investigated Mediation Paths via State Anxiety

**FIGURE 4:**
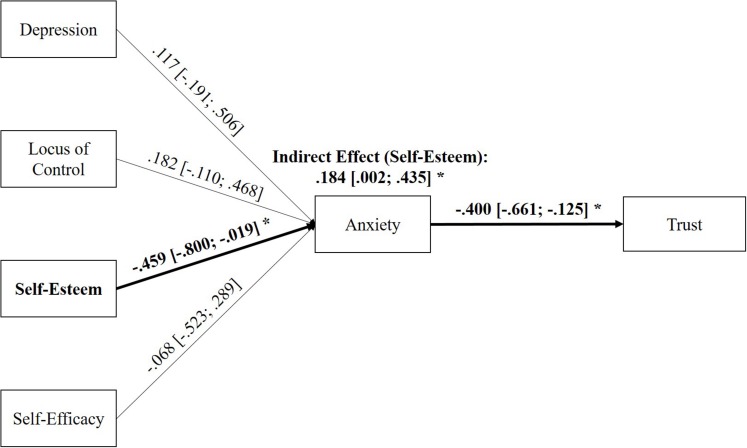
Standardized direct and indirect effects of the personality variables on trust in automation mediated by state anxiety if all personality variables are considered in one path model together. Significant effects (α = 0.05) are indicated by an asterisk. Only the indirect effect from self-esteem on Trust was significant while the indirect effects for the remaining personality variables were not.

In order to test the relative importance of the investigated personality variables, a mediation model considering all traits together was calculated (see Figure 4). The results revealed that only the direct effect of self-esteem on state anxiety remains significant when all investigated personality variables are considered together in one model (β = −0.459 [−0.800; −0.019]). As a consequence, only the indirect effect of self-esteem to trust in automation mediated by state anxiety was found to be significant in the simultaneous path model (β = 0.184 [0.002; 0.435]). While this finding does not restrict the findings of the separate analyses (as multicollinearity in related trait variables is to be expected), it underlines the special importance of lower levels of self-esteem for an enhanced experience of anxiety in contact with automated driving systems leading to decreased levels of trust in such an automated system.

## Discussion

This research tested the hypotheses that depression and personality variables related to depressiveness and self-evaluation influence the experience of anxiety in the face of a newly introduced highly automated driving system. Furthermore, it was investigated, if increased anxiety in this situation is associated with reduced trust in the system and if anxiety mediates the relationships of the investigated personality variables on trust.

### Summary of Results

Taken together, five of the seven hypotheses proposed in this research were substantially supported by the findings of this study (H1, H3, H4, H5, and H7). Additionally, H2 was partly supported. Overall, the reported study provided substantial empirical evidence for a negative relationship of the extent of anxiety when being introduced to an automated system on subsequent levels of trust in automation (H1). Those who experienced more anxiety at the time when they were introduced to a new and unfamiliar automated driving system showed lower trust levels after some interaction with the automated driving system. Furthermore, self-esteem (H4) and self-efficacy (H5) have been found to significantly predict learned trust in an automated system. Most interestingly, these relationships are mediated by the experience of anxiety at the time when the automated system was introduced providing substantial support for a role of anxiety for the formation of trust in automated driving systems. The prediction of trust by anxiety and the associated mediated indirect effects by anxiety were substantially higher than those by positive or negative affect. While there was no direct correlation between depression and trust in automation, a significant indirect effect supports the role of the individual tendency to experience depressive symptoms for trusting a technological system (H2). On the other hand, for LOC neither a direct nor an indirect effect on trust in automation could be found (H6). In the combined path model with all investigated personality traits, the special importance of self-esteem became apparent. Before we discuss our findings in detail, in summation of the reported findings, it can be concluded that anxiety is an important predictor for differences in trust in automation and that an enhanced experience of anxiety is well predicted by personality variables related to feelings of depression and self-evaluation. This finding underlines the importance of individual differences in users in the perception of their capability to use an automated system on the one hand for the experience of emotions when being confronted with unfamiliar automated technology and on the other hand for their subsequent trust in this system. The psychological mechanism supported in the presented study (personality → emotional states → trust in automation) helps to gain a better theoretical understanding for trust formation and calibration processes and provides starting points for the design of measures to facilitate trust calibration during system familiarization and early system use.

### Theoretical Implications

The presented study supports a mediation of the effect of individual differences of users to learned trust after the first interaction with an automated driving system by the individual experience of anxiety when being introduced to the system at first.

#### Role of Anxiety for Trust in Automation

In line with models proposing an influence of emotional states on judgments (Forgas, 1995; Dunn and Schweitzer, 2005), this study provides empirical support for a negative relationship of anxiety to trust in an automated driving system. Trust as an attitude that guides decisions in situations of risk and uncertainty is influenced by the individual level of anxiety in this new and unfamiliar situation. This research enhances the understanding of emotional processes at play in the phase of getting to know and understand a newly introduced automated system. The moderate to high effect size of the relationship between anxiety and trust in automation, explaining 16% of the variance in trust, further highlights the importance of internal situational factors, as proposed in trust in automation models (e.g., Hoff and Bashir, 2015; Kraus et al., 2019b). The role of anxiety for the formation of trust toward a new and unfamiliar automated system underlines the affective facet of trust in automation (e.g., Lee and See, 2004). The psychological mechanism relating the experience of anxiety to trust in automation might be further investigated in future research. Interesting starting points might be processes associated with the subjective representation of risks and dangers in relation to advantages and gains provided by the automated system. Interestingly, the relationship of anxiety with trust in the system was stronger than the relationship of positive or negative affect with trust, which were investigated before (Stokes et al., 2010; Merritt, 2011). This strong relationship of anxiety and trust might be rooted in the theoretical relation between anxiety, the individual disposition to evaluate negative outcomes like risks and losses (risk/loss aversion), and trust (e.g., Thielmann and Hilbig, 2015). Future studies might investigate if the influence of anxiety also diminishes after time like it has been found for positive and negative mood (Stokes et al., 2010).

#### Role of Personality for the Experience of Anxiety and Trust in Automated Technology

Another goal of the presented study was an investigation of the influence of depressiveness and a tendency for negative self-evaluations (self-esteem, self-efficacy, and LOC) in the formation of trust in automation.

In this study, while depressiveness did not directly predict trust in automation, an indirect effect via the experience of anxiety was supported. Participants who experience more depressive symptoms experienced more anxiety before being introduced to an automated driving system. In this regard, the reported association of depression and anxiety generalized to the interaction with the automated system in the study. The higher experience of anxiety in depressed persons might be in part rooted in distorted self-confidence, mood and concentration (e.g., Fried et al., 2016; Fried, 2017; whereas the latter is related to attentional processes) – variables that have in turn been related to the formation of trust in automation (Hoff and Bashir, 2015). While this research provides a first investigation of the role of depression for trust in automation, future studies should further investigate this relationship.

Furthermore, this study supports the role of negative self-evaluations for both the experience of anxiety before being introduced to a new automated system and trust in the system after some initial interaction. The association of self-esteem and self-efficacy to an increased experience of anxiety resembles the findings of studies in other domains (Chamorro-Premuzic et al., 2008; Xiao et al., 2014). For these two personality characteristics (when investigated individually) also the mediation of their effect on trust by state anxiety was significant. People, who perceive themselves to be worthy and capable of performing well in a broad array of tasks, experience a lower level of anxiety when being about to interact with a new automated system. This lower level of anxiety is associated to higher trust in the system subsequently. On the contrary, people with lower regard for themselves also doubt their ability to manage new challenges well and therefore are more scared in unfamiliar situations. This experience of anxiety in turn translates to lower trust in an automated system that has been interacted with for a while. On the other hand, unlike hypothesized, a direct association between LOC and trust in the system was not supported by the data. This could be due to the fact that LOC refers to a general attribution of an outcome, not to its valence (e.g., Ajzen, 2002). In LOC, the attribution who is responsible for an outcome is more important as in self-esteem and self-efficacy. Moreover, when all investigated psychological variables were considered in one model, self-esteem showed the strongest association with anxiety. Self-esteem, when considered alone, explains 36% of the variance in state anxiety. In the reported study, the global evaluation of one’s self-worth was a more prominent predictor for the experience of anxiety and individual differences in trust in automation than all other considered personality variables, also compared to self-efficacy which is related to one’s beliefs to be able to successfully perform in a new task. How people evaluate their own abilities in general thus has far-reaching implications on their experience of anxiety in unfamiliar situations, which in turn mediates the effect of this generalized self-evaluations on the individual level of trust in automation.

Taken together, these findings underline the role of personality characteristics associated with the perception of one’s self-worth and the assessment of one’s capability to perform for the experience of anxiety in the adoption of automated driving systems. These psychological mechanisms are important to be considered in the design and dissemination of automated driving technology.

### Strengths, Limitations and Future Research

This study is the first of its kind to investigate a mediation of personality effects on the evaluation of an automated system by anxiety. An essential strength of this research is that predictors and criteria variables were assessed at different points in time. This allows for an approximation of causality in a correlative design. Also, the proposed direction of effects from personality via states to trust in automation is in line with theory and empirical findings on the associations between personality and emotional states and judgments in general and trust in specific (Schwarz and Clore, 1988; Forgas, 1995; Dunn and Schweitzer, 2005; Forgas and East, 2008). Any other order of these constructs in the mediation process would not be meaningful from a theoretical perspective. Furthermore, this study was conducted in a driving simulator laboratory, which allows for a certain realism and immersion as compared to other set-ups typically used in correlative designs (e.g., online studies). Also, the study used established validated scales for all measurements, which further increases the external validity of the findings.

Besides from these strengths, the presented study comes with some limitations to be considered, and that might be addressed in future studies. First, although most hypothesized relationships were supported by the data, some of the non-significant results might be due to the restricted power of this study and could be investigated in studies with larger samples. Second, the sample consisted in the mere majority of not depressed and subclinical depressed persons. The relationship of depression, anxiety, and trust might be further elevated in persons with a clinical MDD diagnosis. Future studies might further investigate the reported relationships in clinical samples. Third, the included psychological variables only reflect a selection of theoretically relevant variables for the emergence of individual differences in situational trust appraisals in the interaction with automated (driving) systems. Also, in this regard, future studies could investigate the relationship of other emotional states besides positive, negative affect, and anxiety to trust in automation. Furthermore, potential moderating variables of the mediation cascade from traits via emotional states to trust or automation-related behavior might be investigated. Additionally, future studies could manipulate the relevant system or situational characteristics that trigger the experience of anxiety to better understand the relationship of anxiety and trust in automated systems. For example, the perception of risk and uncertainty of using an automated system might be manipulated in future studies. It has to be noted that the reported findings neither restrict the hypothetical relevance of LOC nor that of positive and negative affect for the formation of trust as the theoretically expectable directions of effects could be observed and might only not been significant due to this study’s relatively small sample. Whatsoever, as the remaining effects were significant with this sample size, at least it might be concluded that the predictive power of those variables with supported relationships seems to be somewhat higher under the circumstances of the reported study.

### Practical Implications

The relationship of anxiety and trust in automation underlines the importance of paying respect to users’ emotional states, feelings of uncertainty and risk perception in people, who initially get to know an automated system and interact with it for the first time. As a consequence, practitioners might pay attention to consider in which situations and in which mindset users are when they are first introduced to an unfamiliar system. For example, it seems worthwhile to reduce the perception of uncertainty and risks in this situation and rather provide reassuring information in order to reduce anxiety and increase initial trust in the system. Thereby it should be kept in mind that the goal of the process of introducing the user to a new system is always a calibrated level of trust rather than maximizing trust irrespective of the actual capabilities of the system.

Also, the reported relationships of individual feelings of depression and negative self-evaluations in regard to one’s worth or capability to effectively manage new tasks with increased anxiety levels and decreased trust in automated systems allow for the derivation of implications for practice. The reported study findings point into the direction of individualized communication and training of system functionality as well as personalized design of user interfaces in orientation to personality differences to facilitate an appropriate level of trust in different user groups. For example, people with lower self-esteem and lower self-efficacy could be provided with a specific driver training that bolsters against anxiety in the first (independent) use of the automated driving system. Such training could explicitly assess which unrealistic fears exist and address them by providing factual information about the capabilities and functioning of the respective system. On the other hand, persons with high levels of self-esteem and self-efficacy might tend to experience low levels of anxiety potentially leading to overtrust in the system. To prevent them from using the system in situations it is not designed for a personalized driver training might stress potential dangers and risks in the interaction with the automated driving system under consideration and thereby enhancing a more realistic assessment of their own and the system’s abilities. Regarding the design of automated systems, user groups with different levels of positive or negative self-evaluation will likely react very differently to certain information in different phases during system familiarization. Practitioners might therefore carefully balance out the provided information about system functioning and system reliability in automated driving systems under consideration of both their costumers’ individual level of anxiety and their self-assessment of the capability to use the system adaptively. Above this, similar to the mentioned personalized driver training, personalized design of user interfaces might be useful for users with different levels of a positive or negative self-evaluation to facilitate individual trust calibration.

The role of depressive symptoms for the experience of anxiety and the formation of trust in automated systems underlines that besides subclinical personality traits also clinical diagnoses should be considered in the dissemination phase of driving automation technology. While this is on the one hand important for manufacturers it is also essential to be addressed on a societal and legislative level in the face of the growing prevalence of MDD. The reported findings of an association of the experience of depressive symptoms and anxiety in turn associated with decreased levels of trust in automation are of special interest as depressive symptoms and the intake of respective medication have been related to negative driving behavior like risky driving (e.g., McDonald et al., 2014), increased reaction times (e.g., Bulmash et al., 2006) and increased risk of accidents (e.g., Bulmash et al., 2006; Hilton et al., 2009; Cameron and Rapoport, 2016), for which driving automation might provide a solution. Therefore, people with depression symptoms are among those who could benefit most from driving automation, while at the same time being especially prone to the experience of anxiety in the face of unfamiliar and potentially risky situations (like the interaction with a new automated driving system). This underlines the importance of explicitly addressing their fears both on an individual and societal level. Additionally, for example, the acquisition of automated driving aids might be funded by governments or health insurances to provide a broader access to automated driving aids for depressed persons. Hereby, it should be carefully taken into consideration to not discriminate against people with MDD but rather to empower them to fully benefit from the significant alleviation by this technology for their mobility.

### Conclusion

Taken together, this research underlines the importance of considering anxiety and other emotional states in trust formation when people first get to know automated systems. Furthermore, the experience of anxiety when being introduced to the new technology was found to be rooted in the individual tendency to experience depressive symptoms and have negative self-evaluations. While a certain degree of anxiety is a normal and adaptive reaction in the face of risky and potentially dangerous new technology, in order to facilitate a calibrated level of trust, it should be taken care that anxiety levels do neither fall below nor exceed the actual level of dangers and risks posed by this new technology as otherwise, this might lead to over- and distrust. To facilitate a calibrated level of trust, the investigated psychological mechanisms in this study underline that both situational and personality characteristics associated with an experience of anxiety in the face of driving automation should be considered.

## Data Availability Statement

The datasets generated for this study are available on request to the corresponding author.

## Ethics Statement

The study was carried out in accordance with the Declaration of Helsinki. Ethical review and approval was not required for the study on human participants in accordance with the local legislation and institutional requirements. The patients/participants provided their written informed consent to participate in this study.

## Author Contributions

JK generated the project idea, collected the data, performed the analyses, and led the manuscript write-up. DS assisted with collecting the data, data analyses, and project write-up. E-MM assisted with the data analyses and project write-up. MM assisted with generating the project idea and project write-up. MB assisted with the project write-up.

## Conflict of Interest

The authors declare that the research was conducted in the absence of any commercial or financial relationships that could be construed as a potential conflict of interest.
